# Graph network analysis of immediate motor-learning induced changes in resting state BOLD

**DOI:** 10.3389/fnhum.2013.00166

**Published:** 2013-05-15

**Authors:** S. Sami, R. C. Miall

**Affiliations:** Behavioural Brain Sciences Centre, School of Psychology, University of BirminghamBirmingham, UK

**Keywords:** fMRI, resting state, graph analysis, complex networks, motor learning

## Abstract

Recent studies have demonstrated that following learning tasks, changes in the resting state activity of the brain shape regional connections in functionally specific circuits. Here we expand on these findings by comparing changes induced in the resting state immediately following four motor tasks. Two groups of participants performed a visuo-motor joystick task with one group adapting to a transformed relationship between joystick and cursor. Two other groups were trained in either explicit or implicit procedural sequence learning. Resting state BOLD data were collected immediately before and after the tasks. We then used graph theory-based approaches that include statistical measures of functional integration and segregation to characterize changes in biologically plausible brain connectivity networks within each group. Our results demonstrate that motor learning reorganizes resting brain networks with an increase in local information transfer, as indicated by local efficiency measures that affect the brain's small world network architecture. This was particularly apparent when comparing two distinct forms of explicit motor learning: procedural learning and the joystick learning task. Both groups showed notable increases in local efficiency. However, a change in local efficiency in the inferior frontal and cerebellar regions also distinguishes between the two learning tasks. Additional graph analytic measures on the “non-learning” visuo-motor performance task revealed reversed topological patterns in comparison with the three learning tasks. These findings underscore the utility of graph-based network analysis as a novel means to compare both regional and global changes in functional brain connectivity in the resting state following motor learning tasks.

## Introduction

The combination of resting state neuroimaging methods with motor learning paradigms has ushered in a new era to the investigations of adult brain plasticity. Until recently neuroimaging paradigms examining motor learning were almost exclusively investigated during the execution of a learning task. This has generated a wealth of data showing rapid neural changes occurring during the execution of the learning task. Although the vast majority of these studies were investigated with fMRI, other techniques such as diffusion weighted imaging have shown that long term motor practice can induce structural changes in both gray (Maguire et al., 2000) and white matter (Scholz et al., 2009; Johansen-Berg, 2010; Tomassini et al., 2011). So, given that learning a new skill alters both functional and structural brain networks, one key unanswered question is how the rapid functional changes seen in task related activity contribute to sustain longer term changes in structure or function i.e., in essence the relationship between short-term and long term motor memory. While it has been previously speculated that resting state functional networks may hold at least a partial answer to this question (Miall and Robertson, 2006; Albert et al., 2009; Ma et al., 2010), it was not until recently that such a link has been provided (Taubert et al., 2011; Vahdat et al., 2011).

However, many questions about the very nature of functional resting states remain unanswered (Deco et al., 2011). Ever since Biswal and colleagues measured spontaneous activity over the motor cortex there has been a great interest in resting state networks (Biswal et al., 1995). “Resting state activity” usually measures endogenous and spontaneous rhythms and can be considered low frequency fluctuations in the BOLD signal. It has now been established that resting state-brain networks (RSNs) are highly reliable, showing reproducible traits over time, over subjects and across testing sessions, as well as having a strong association to task-related activation patterns (Smith et al., 2009). Recent studies have investigated the functional relevance of resting state networks by striving to link changes in RSNs with known functionally active task-related networks. One of the first studies was by Albert et al. (2009) investigating the effect of a visuo-motor learning task on resting state BOLD. They found that the fronto-parietal and cerebellar networks are particularly engaged following learning, highlighting that functional changes seen in resting state immediately following motor training are representative of changes generally seen during motor learning task performance. Moreover, this comparative approach has given us an additional insight into RSNs, highlighting common characteristics between brains regions that share a common function (Smith et al., 2009). The comparisons between RSN and task-based network modulations has been largely achieved through the use of novel neuroimaging techniques like seed-based correlations and ICA, and have allowed the categorization of further functional sub-networks (Van den Heuvel and Hulshoff Pol, 2010).

Even though a number of key networks have been identified through ICA little is known about their network properties. More recently, graph theoretical network analysis has provided a novel approach to identify biologically plausible network architectures and this could provide insight into organizational rules as well as the processing properties of these networks following learning (Heitger et al., 2012).

Graph analysis of neuroimaging data is still a very new technique. Until now the most common use of graph-based analysis of resting fMRI data, has been to characterize normal functional connectivity at rest, and to examine differences in brain networks in healthy individuals compared to patients with neurological disorders (Liu et al., 2008; Lynall et al., 2010). Most recently a few studies have utilized graph analysis of neuroimaging data related to motor learning. Bassett et al. (2011) looked at dynamic changes following a simple motor learning task focusing on modular network changes only, while Heitger et al. (2012) looked at a more complete set of graph analytic measures investigating motor learning in a task based experiment. Given the paucity of work on resting state graph based analysis following motor learning, we were interested in whether these techniques can usefully complement more common ICA-based approaches. Here we utilize this graph analytic approach to compare immediate changes induced in the resting state following four motor tasks. Two groups of participants performed a visuo-motor target-tracking task with one group adapting to a transformed relationship between joystick and cursor. Two further groups were trained in either explicit or implicit procedural sequence learning. Based on our previous ICA results (Albert et al., 2009) and on a recent meta-analysis of the motor learning literature (Hardwick et al., 2012), we hypothesize that the visuo-motor tasks will show significantly stronger cerebellar activity while the procedural sequence-learning tasks will show more widespread cortical activity.

## Materials and methods

We used resting state BOLD signal data from four motor tasks, two variants of a sequence-learning task requiring rapid finger button presses in a learned sequence, and two variants of a target-reaching task using a joystick. Both sequence-tasks were designed to induce learning, one explicit and one implicit; one of the visuo-motor tasks was a learning task, the other a non-learning control task. In each we compared resting activity before and after the learning period.

### Tasks 1 and 2: sequence learning

#### Participants

Two groups of twelve healthy individuals participated in either an explicit (task 1) or implicit (task 2) version of the serial reaction time task (SRTT; Robertson, 2007). All participants were right handed, as confirmed by the Edinburgh handedness questionnaire. All participants (mean age 23.6 ± 5.2 years) gave written informed consent, and received either cash or credit for their participation. Participants recalling more than four items of the sequence were excluded from the implicit condition. All the participants were instructed to respond as quickly and as accurately as possible to the target location by a button press. Moreover, instructions to participants differed depending on which group they participated in. The implicit participants were unaware of the underlying sequence; while the explicit participants were aware of the existence of a sequence that was highlighted by a different color than the embedded random sequences. The two tasks were equalized in terms of the testing block size to avoid durational performance effects. The task lasted approximately 10 min for the explicit group while it was only slightly longer (~by 2 min) for the implicit group. The local ethics committee at the University of Birmingham approved the experiment.

#### Procedure

Participants were scanned with a 3T Philips Achieva MRI scanner as they completed a fixed set of tasks. First they viewed a dynamic point light display of human body movements, as a dummy task (Albert et al., 2009). They were then instructed to lie still with their eyes open while fixating on cross displayed in the middle of the screen during the initial rest scan which lasted for 10 min. An explicit or implicit procedural learning SRTT task was then issued for approximately 15 min, dependent upon individual reaction times. Participants responded with their right hand using a 4-button response box. The dummy task was then repeated for 5 min. Finally participants remained for a second 10 min rest scan conducted ~5 min after the end of the SRTT task.

### Tasks 3 and 4: visuo-motor learning

Data from a previously reported study have been reanalyzed here. Details of the procedures are found in the original report (Albert et al., 2009). In summary: two groups of twelve individuals participated in one of two visuo-motor tracking tasks. Participants used an MR compatible joystick to control a cursor with their non-preferred left hand. For the test group (task 3) there was an angular displacement between target and cursor accumulating by 10° every min for 10 min, reaching a maximum of 90°, while for the control group (task 4) there was no such displacement, and the movements of joystick and cursor were congruent. As in tasks 1 and 2 each of the visuo-motor tasks were interleaved between the two rest sessions and had the same dummy task preceding every rest period acquisition.

#### Imaging parameters

For all 4 experiments, scans were conducted at the Birmingham University Imaging Centre (BUIC), University of Birmingham, Birmingham, UK; the experiments were approved by the University's local ethical panel, and all participants gave written informed consent. The MRI unit was a 3 Tesla Philips Achieva scanner (Koninklijke Philips Electronics N.V., Eindhoven, Netherlands). Each participants had a high-resolution T1-weighted structural scan where the *TR* = 8.4 ms, *TE* = 3.8 ms, flip angle = 8° and FOV = 232 × 288 × 175 mm). In all functional scans the *TR* = 2800 ms, *TE* = 35 ms, and flip angle = 85°. An 8 channel (SENSE factor 2) head coil was used. EPI volumes consisted of forty-nine 96 × 96 axial slices of 2.5 × 2.5 × 3 mm voxels. Using an FOV of 240 × 147 × 240 mm, the entire cerebral cortex and cerebellum were covered.

#### Image pre-processing

All data were motion-corrected and normalized to a standard template using the statistical parametric mapping software (SPM8; Friston et al., 2006). Pre-processing included regression of motion parameters, nuisance signals, and global signal, followed by band-pass filtering at 0.01–0.1 Hz to isolate the low-frequency fluctuations characteristic of resting connectivity. Data was then parcellated into 116 regions using the Automatic Anatomical Labeling (AAL) atlas as implemented by IBASPM (Tzourio-Mazoyer et al., 2002; Alemán-Gómez et al., 2006). This resulted in an averaged fMRI time series for 116 regions (nodes) for each subject, which were used for subsequent graph network analysis (see Figure 2).

#### Network graph construction

**Figure 1:**
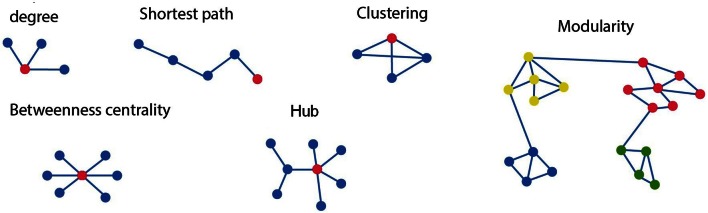
**Schematic diagram of different graph measures.** In the upper row, starting from left, shows the *degree*, which is the number of edges connected to each node. As indicated here the red node has a *degree* of three. The *clustering coefficient* of a node is given as a ratio of its neighbors that are also linked to one another. The red node is connected to all three possible neighboring nodes but forming only 2 of the 3 possible closed triangles. Hence the *clustering coefficient* for this node is 2/3. The *path length* of the red node is four as minimum number of connections between the red node and final blue corresponds to four. On the bottom row *betweenness centrality* is the ratio of all shortest paths that pass through and from a node. The connector *hub* is shown in red as connections between the nodes with the highest degree. The graph at the right consists of four different *modules*, or *clusters* specified by the different node color. *Modularity* refers to the existence of clusters of nodes with connections which are more densely connected to other nodes within the same module than to nodes outside the module.

**Figure 2:**
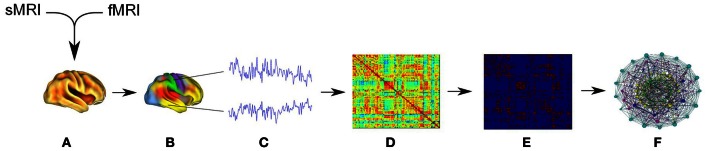
**The diagram represents data analysis workflow. (A)** Initial data pre-processing and co-registration of structural and functional data. **(B)** Application of parcellation scheme anatomical template image to each individual fMRI dataset creating a regional mean fMRI time series. **(C)** Wavelet analysis was used to bandpass filter the regional time series and to estimate frequency-specific measures of functional connectivity between regions. **(D)** The creation of functional connectivity adjacency matrices **(E)** The adjacency matrices were thresholded and binarized then these undirected graphs were used for the creation of **(F)** network topological metrics were further evaluated by statistical testing.

To create network graphs for every participant (see Figure 1), we used the Matlab-based Connectivity Decoding Toolkit (Richiardi et al., 2011). This software applies the outcome of the widely adopted IBASPM structural atlas to form a functional atlas by averaging the time series data for each region. It then performs a discrete wavelet transformation on the averaged time series data, filtering it into four separate frequency sub-bands. Here we adopted the use of a standard sub-band (0.06–0.1 Hz), which has been widely used for resting state analysis. In practice this sub-band has been shown to effectively filter out physiological noise in upper frequencies, and avoids measurement errors connected with lower frequencies (Fornito et al., 2010; Richiardi et al., 2011). For functional connectivity between the 116 parcellated regions, the Pearson correlation was computed between all pairs of node time series to generate a 116 × 116 correlation matrix (i.e., the adjacency matrix, *Aij*) for each subject (see Figure 2). The adjacency matrix represents a very densely connected network that makes it difficult to test the reliability of the connections. For simplicity the adjacency matrix is thresholded and further binarized to maintain only the most reliable connections (Rubinov and Sporns, 2011). In this study we adopted five thresholds of *r* = 0.3, 0.4, 0.5, 0.6, and 0.7.

#### Network measures

Once the binary graphs were constructed, the Brain Connectivity Toolbox (Rubinov and Sporns, 2010) was used to calculate the network measures. All network measures used thresholded and binarized graphs with the exception of the *strength* measure, which can only be applied to complete graphs. Although a large number of measures can be used (Rubinov and Sporns, 2010), in this study we used 10 selected measures of the thresholded binary graphs, choosing those most consistent across the literature, yet allowing us to capture the important features of the complex graphs. Network based measures used in this study include *degrees, hubs, characteristic path length, clustering coefficients, local and global efficiency, small worldness, betweenness centrality*, *modularity, and participation coefficients*.

#### Topological properties of the network

***General measures of connectivity.*** One of the fundamental measures widely used in graph analysis is that of connectivity degree. The degree *D*_*i*_ of a node/region *i* is characterized as the total number of edges connecting that node/region to its neighbors (see Figure 1). An increase in level of global network interaction for a given region is signified by increase in degrees. Nodes with the highest degrees can also be signified as hubs. The degree *D* of a graph B is the mean of the degrees for the total number of nodes in the graph (Heitger et al., 2012).

$$D=\frac{1}{N}\sum_{i\epsilon B}D_{i}$$

Another measure of global connectivity is *strength* (*S*_*i*_). For a given region this is defined and computed as the sum of weights *w*_*ij*_ (connection density) of all the connections of a region/node *i*, providing information on the total level of weighted pair-wise correlations of the region/node. In mathematical terms:

$$S_{i}=\sum_{j\epsilon N}w_{ij}$$

In turn, the total connection *strength* S of the graph was computed as the mean of *S*_*i*_ for all nodes (Sporns, 2011; Heitger et al., 2012).

*Path length* provides information on global information transfer efficiency, as a shorter path would allow for the more rapid distribution of information between brain regions, with shorter paths entailing a greater prospect for integration (see Figure 1).

The mean shortest path length *L*_*i*_ of a node *i* is:

$$L_{i}=\frac{1}{N-1}\sum_{i\neq j\epsilon B}L_{i,j}$$

The characteristic path length *L* of a network is the mean of the shortest path length between the nodes (Sporns, 2011; Heitger et al., 2012).

Furthermore, *global efficiency* of a network is also associated with path length and generally defined as the mean of the inverse shortest path length (Latora and Marchiori, 2001).

***Region based measures of functional connectivity.*** Densely interconnected groups of nodes are known as clusters within the network. These clusters can be defined on either a regional or network level (see Figure 1). The *clustering coefficient* of a node or region *C*_*i*_ is a ratio between the numbers of existing edges among the node's neighbors divided by the total number of all the regions possible edges:

$$C_{i}=\frac{R_{i}}{D_{i}(D_{i}-1)/2}$$

*R*_*i*_ is the total number of connected pairs between all neighbors of node *i*. On a network level the clustering coefficient *C* is defined as the mean of the clustering coefficient of all nodes (Sporns et al., 2004; Sporns, 2011; Heitger et al., 2012).

*Local efficiency E*_*i_loc*_ of a node *i* is linked to the clustering coefficient and is defined as:

$$E_{i\_loc}=\frac{1}{v_{i}(v_{i}-1)}\sum_{j,v\epsilon H_{i}}\frac{1}{L_{j,v}}$$

where the sub-graph *H*_*i*_ represents nodes that are connected to the node *i* and in which *L*_*j*, *v*_ is the minimal number of edges connecting node *j* and node *v* (similar to shortest path description) and *vi* (similar to *N*). *E*_*i_loc*_ discloses how efficient the communication is between node *i* and its neighbors. The mean local efficiency of a graph, is merely the mean of the local efficiency of all the nodes in the graph (Sporns, 2011; Heitger et al., 2012).

***Small-world brain connectivity.*** Small-world networks can be described as networks that have approximately the same characteristic path length as random networks, yet are notably more clustered than random networks, (Watts and Strogatz, 1998), Formally:

$$\begin{array}{l} \gamma =C^{\text{real}}/C^{\text{rand}}>1\\ \lambda =L^{\text{real}}/L^{\text{rand}}\approx 1 \end{array}$$

where the *L*^real^ and *C*^real^ are the characteristic path length and clustering coefficient of the real network, the *L*^rand^ and *C*^rand^ are the mean characteristic path length and clustering coefficient of an comparable random network, i.e., a random network that has similar graph characteristics in terms of size and edges as the real network (Maslow and Sneppen, 2002; Sporns et al., 2004). The *small worldness* coefficient is defined as a ratio σ = γ/λ, where values of sigma greater than 1 can be considered small world (Sanz-Arigita et al., 2010).

Measures founded on the notion of *centrality* are described as the most important nodes that contribute to the shortest paths inside a network and as a result act as central controls of information flow (Rubinov and Sporns, 2010). A commonly adopted *centrality* measure is *betweenness centrality X*_*i*_ of a node *i*, is defined as:

$$X_{i}=\frac{1}{\left(N-1)(N-2\right)}\sum_{\begin{array}{c} f,j\epsilon G\\ f\neq j,f\;\neq i,j\;\neq i \end{array}}\frac{Y_{fj(i)}}{Y_{fj}}$$

in which *Y*_*fj*_ is the total sum of shortest paths connecting nodes *f* and *j* and *Y*_*fj*(*i*)_ is the total sum of shortest paths linking nodes *f* and *j* that go through node *i*.

The principle nodes often referred to as hubs can also be described as those nodes with the greatest *betweenness centrality* in a complex network (He et al., 2007; Shu et al., 2009; Rubinov and Sporns, 2010).

***Modularity.*** A module is defined as a sub-network of highly inter-connected nodes that are comparatively sparsely linked to nodes in other modules (see Figure 1). Modularity in brain networks is associated with densely connected neighboring functional or anatomical cortical areas or communities, while connections between modules tend to be comparatively long distance (Meunier et al., 2010). The modularity detection algorithm we used was based on the Louvain method (Blondel et al., 2008) and visualized with a circular diagram. This is an efficient method for identifying modular structures. This is based on an algorithm that maximizes modular detection by iterative searching over the possible divisions of a network until modularity for a given module cannot be further improved.

The *modularity* measure: *Q* is originally defined as an unweighted and undirected network that is partitioned into sub-networks (Newman, 2004; Meunier et al., 2010)

$$Q=\frac{1}{2\alpha }\sum_{Z\epsilon \;P}\times \sum_{j,D\epsilon B_{i}}\left(A_{ij}-\frac{k_{i}k_{j}}{2\alpha }\right)$$

where *A* is the adjacency matrix of the network; α is the total number of edges; *ki* and *kj* are the degrees of node *i* and *j*. The index *Z* runs over the modules of the community or partition *P*. *Modularity* compares the number of links between the numbers of possible connections for all pairs of nodes within a sub-network, against the number of such edges for a corresponding random graph.

Following the optimal partitioning of a network into modules, individual nodes can be ascribed to characterize their impact for within and between -modular transfer of information (Guimerà and Amaral, 2005; Meunier et al., 2010). The *participation coefficient* of a given node is the proportion of edges linking it to nodes in other modules.

$$\Omega_{j}=1-\sum_{W=1}{\left(\frac{\beta_{jc}}{\beta_{j}}\right)}^{2}$$

where β_*jc*_ is the number of links of node *i* to nodes in module *W* and β_*j*_ is the degree of node *i*. If all the edges of node *i* are distributed within their module, then β_*jc*_ = β_*j*_ and the participation coefficient Ω_*j*_ is 0. However, if all the connections of node *i* are distributed between the rest of the modules, Ω_*j*_ approaches one (Guimerà and Amaral, 2005).

#### Statistical analysis

We tested for significant differences between the pre- and post-motor task RSN measurements using the non-parametric Wilcoxon statistical hypothesis test when comparing related groups, at identical thresholds for each network measure. Additionally, we corrected for multiple comparisons to identify within group (corrected for the 116 nodes) and between-group differences (corrected of the four groups) across all network measures (Zalesky et al., 2010).

## Results

Behavioural results indicate that motor performance significantly improved across all three learning tasks [visuo-motor data was reported in Albert et al. (2009)]. Hence learning was induced in each case, but the task differences imply that we would expect different network changes underlying this change in performance. The performance in the non-learning visuo-motor control group did not change (Albert et al., 2009).

For the SRTT both groups showed procedural learning following the training exposure phase of the SRTT task. Comparing pre- and post-training for the implicit task, there was a significant reduction in reaction times (*p* <0.05), while the explicit task showed a greater difference (*p* < 0.01). There was also a marked difference between the performance of these two groups, with significantly reduced reaction times for the explicit group (*p* = 0.01). This is an expected outcome due to the more rapid sequence acquisition of participants with explicit awareness (i.e., in the “explicit group”).

### Global changes in *strength*

**Figure 3:**
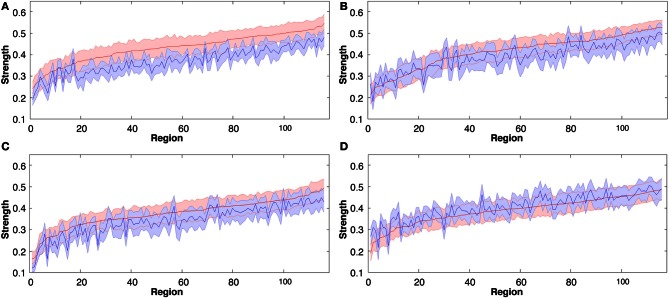
**Group mean connectivity *strength* for each of the 116 regions before and following all four tasks, rank ordered by mean regional *strength* measured in rest 2 (pre-task rest 1 data are shown in blue, post-task rest 2 data in red).** The solid line represents the mean while shaded area indicates SEM. **(A)** Explicit SRTT task 1, **(B)** Implicit SRTT task 2, **(C)** Visuo-motor learning task 3, and **(D)** Visuo-motor performance task 4.

To define global changes in the resting state networks after learning we measured the correlation coefficient calculated on RSN-specific low frequency components of the BOLD signal. For each of 116 anatomically defined brain regions, we estimated the *strength* (Rubinov and Sporns, 2010) of its functional connectivity to the rest of the brain in each individual dataset. In all 3 of the motor learning tasks functional connectivity *strength* was significantly greater in the 2nd rest period post learning (*p* < 0.001; see Figures 3A–C). In contrast overall strength of connectivity was significantly reduced in the visuomotor performance task (*p* < 0.01; Figure 3D). *Strength* also varied widely over different brain regions, as indicated by overall ranking of strength across the 116 regions, and by the local differences in the amount of change in strength between the two rest sessions separated by learning (as indicated by the jagged pre-learning ordered data in blue, in Figure 3, compared to the red post-learning data).

### Local changes in *strength*

For the sequence learning tasks (task 1 and 2), the global changes in *strength* also showed specific local network changes that were persistently higher in the frontal and visual regions for explicit SRTT task contrasted to the implicit SRTT task (*p* < 0.01). Given the different nature of visuo-motor rotation tasks (task 3 and 4) we expected different network responses; indeed, in the learning group (task 3) the most significantly affected nodes were the amygdala and the hippocampus (*p* < 0.01), while for the performance group, there were no significant effects in these brain areas.

To complement these results based on analysis of continuous *strength* measures of association between regions, we also measured the topological properties of the binary (unweighted and undirected) graphs derived by thresholding the Pearson's correlation coefficient of the individual functional connectivity matrices. At each threshold we compared the observed values of *degrees, correlation coefficients*, and *path length* in brain networks to their distributions in comparable random graphs with the same number of nodes and degree distributions.

### Global changes in *degrees*

**Figure 4:**
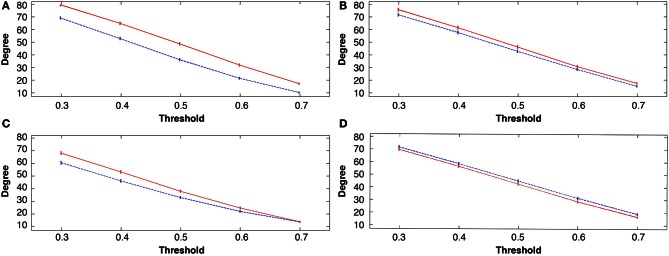
**Group mean *degree* connectivity at all costs before and following the four tasks, in all figures rest 1 (pre-task in blue) and rest 2 (post-task in red); (A) explicit SRTT task 1; (B) implicit SRTT task 2; (C) visuo-motor learning task 3; (D) visuo-motor performance task 4.** Mean is represented by the solid and dotted lines while vertical bars indicate SEM.

All three learning groups showed a significant increase in *degrees* after learning, at all costs or threshold levels (see Figures 4A–C). For the explicit SRTT task 1, *degrees* were the most significantly increased over all costs (*p* < 0.0001) this also indicates a large effect size. This was followed by the visuo-motor learning group (task 3), while the implicit sequence group (task 2) showed the least significant increase across costs among the three learning conditions. In contrast, the visuo-motor performance group (task 4) showed a significant decrease across all 4 cost levels (*p* < 0.001; see Figure 4D).

**Figure 5:**
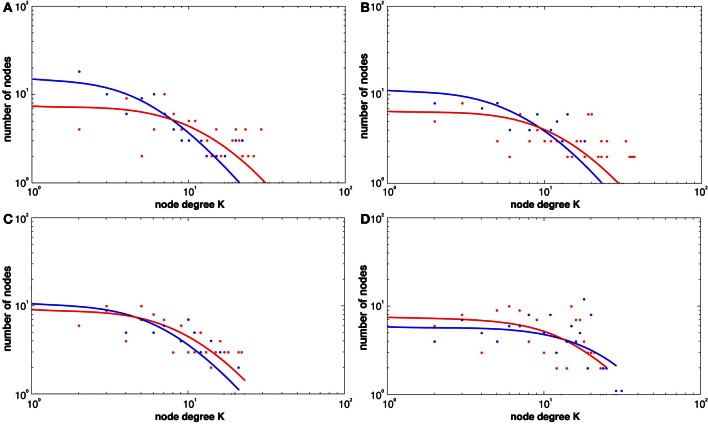
**Histogram degree distributions before and following the four tasks at a threshold of *r* = 0.6 for both REST1 (blue) and REST2 (red), (A) explicit SRTT (B) implicit SRTT task (C) visuo-motor learning task (D) visuo-motor performance task.** The figure shows higher probability of high-degree network hubs following learning (red).

Figure 5 shows the broad scale *degree* distributions consistent with the existence of *hubs*. The figure also highlights the increase in hubs only in the learning groups (see Figures 5A–C) while the visuo-motor performance group (task 4) showed a decrease (see Figure 5D).

### Local changes in *degrees*

For task 1 the main nodes showing significant increases in *degrees* were in the frontal orbital cortex including inferior triangular middle occiptal gyrus (*p* < 0.01), which also showed significant increases in *strength*. However, unlike *strength*, for *degrees*, the right superior partietal gyrus also showed a significant increase in the implicit SRTT task (task 2). Although a similar overall pattern of global increase in degrees was seen in the visuo-motor learning task (task 3; *p* < 0.001) the most pronounced local effects were in entirely different regions. The significantly affected nodes include the right amygdala and left cerebellum (lobule III) (*p* < 0.01) while the visuo-motor adaptation performance group (task 4) showed a significant decrease over the left cerebellum and basal ganglia (*p* < 0.01).

### Global changes in *local efficiency*

**Figure 6:**
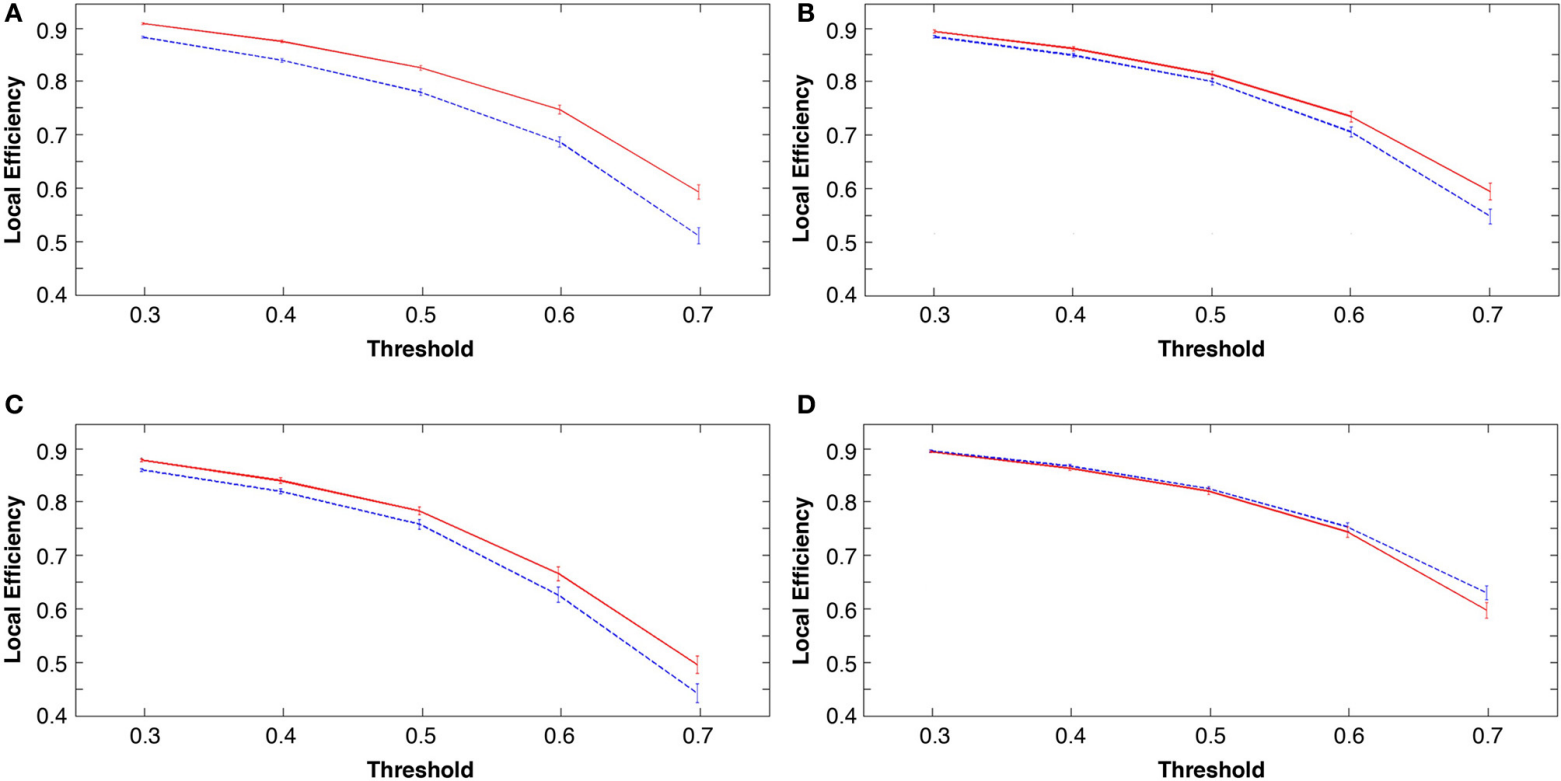
**Group mean local efficiency at all costs before and following the four tasks, in all figures rest 1 (pre-task in blue) and rest 2 (post-task in red). (A)** explicit SRTT **(B)** implicit SRTT task **(C)** visuo-motor learning task and **(D)** visuo-motor performance task. Group mean is represented by the solid line while vertical bars indicate SEM.

The measure of *local efficiency* showed similar post-learning increases across all costs in the learning groups (tasks 1, 2, and 3; *p* < 0.001; see Figures 6A–C). The visuo-motor performance group (task 4) consistently showed a decrease in *local efficiency* in rest 2 across costs (Figure 6D). However, these decreases were non-significant (*p* > 0.05).

### Local changes in *local efficiency*

**Figure 7:**
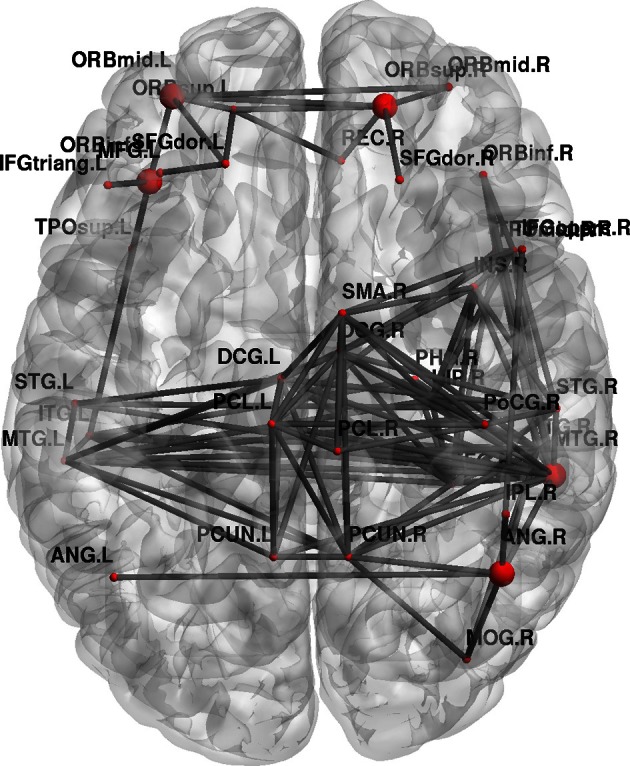
**Change in brain network graphs for regional local efficiency at a threshold of *r* = 0.6 following the explicit SRTT task.** The highlighted regions represent significant nodes with more locally efficient communication in REST 2 compared with REST 1.

**Figure 8:**
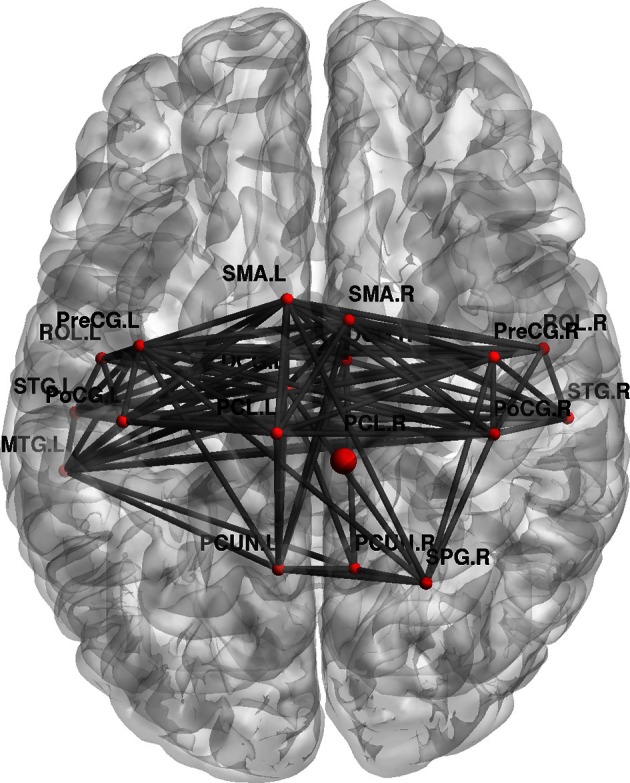
**Change in regional local efficiency following the implicit SRTT task.** The format is the same as in Figure 7.

**Figure 9:**
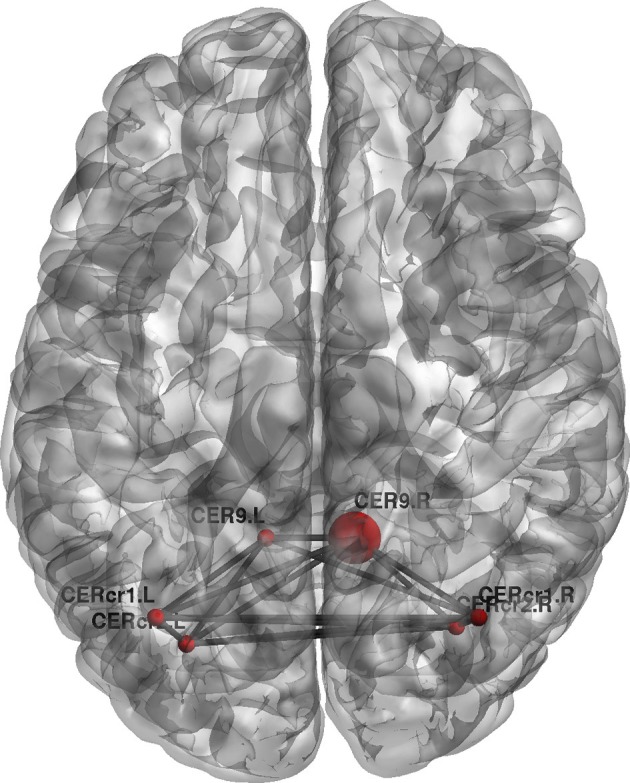
**Change in regional local efficiency following the visuo-motor adaptation learning task.** The format is the same as in Figure 7.

**Figure 10:**
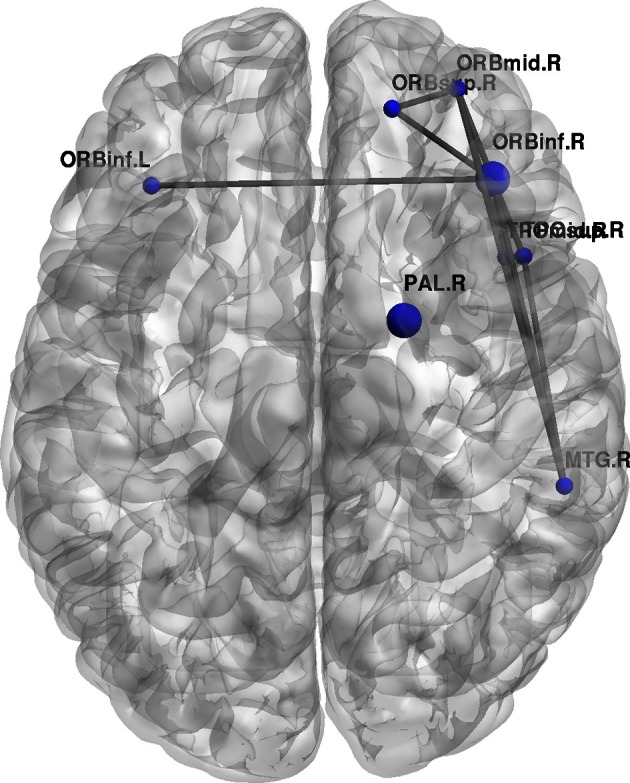
**Change regional local efficiency following the visuo-motor performance task.** The format is the same as in Figure 7; the figure here represents significant decreases in local efficiency between the two rest conditions are highlighted in blue.

Furthermore, topological brain network images highlight the fact that different anatomical networks are affected by the different tasks. The explicit SRTT group (task 1) showed significant increases in *local efficiency* (*p* < 0.05) in the frontal orbital regions and the right angular gyrus and the right medial temporal cortex while the implicit group (*p* < 0.05) (task 2) showed increases in the left precentral gyrus, SMA and the thalamus (see Figures 7, 8). The opposing effects on *strength* seen between the visuo-motor learning and performance groups (task 3 and 4) were also evident for this measure of *local efficacy*: the learning group (task 3) revealed significant increase the right cerebellum (*p* < 0.05) (lobule 9) (see Figure 9), in the left caudate nucleus of the basal ganglia and the left hippocampus (*p* < 0.05), while the performance group (task 4) revealed significant decreases in the right inferior parietal (*p* < 0.01; see Figure 10).

### Global changes in *path length*

Another measure that showed significant decreases across all costs for all learning conditions was *path length* (*p* < 0.001), while the performance group (task 4) showed a significant increase (*p* < 0.001) across all thresholds except at the threshold *r* = 0.6 which showed a more subtle increase (*p* < 0.05).

### Local changes in *path length*

Here the explicit SRTT group (task 1) showed significant and widespread regional decreases in *path length* in the orbital frontal regions, left inferior triangular gyrus, right post central gyrus, left middle occipital cortex, right basal ganglia, and right cerebellum crus II (*p* < 0.05). The implicit SRTT group (task 2) showed increased effects *path length* in the left hippocampus and the left parahippocampus (*p* < 0.05). The visuo-motor learning group (task 3) showed decreases in the precunus, the left amygdala, and the cerebellum while there was also a single increase in the left inferior opercular frontal cortex (*p* < 0.05). The performance group (task 4) did not show any significant changes in *path length* at the node level despite a significant overall increase.

### Changes in *small worldness*

In order to calculate the small worldness coefficient, sigma, we also calculated the clustering coefficient for all the four tasks this produced near identical results to the local efficiency measure (see above). An additional measure that is required for the calculation of small worldness is path length (see above).

**Figure 11:**
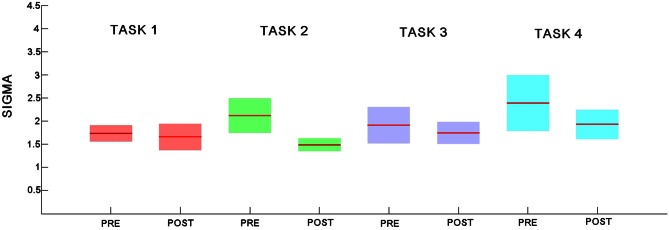
**Small world coefficient sigma for the pre- and post-task rest networks for each of the 4 experiments.** Explicit SRTT task 1: red (pre-task on left and post-task on right); implicit SRTT task 2; green, visuo-motor adaptation learning task 3; purple, visuo-motor performance task 4; blue, Group mean is represented by the solid line while shaded areas indicate SEM.

At a global level, all measures of functional networks expressed some key organizational properties consistently across both groups. All resting state networks including pre task networks showed small world characteristics. At each cost level in the small-world regime, we sampled 1000 random graphs and estimated the mean and SD of each parameter so that we could then calculate. *Small worldness* did not show any significant change (*p* > 0.05; see Figure 11).

### Global changes in *betweenness centrality*

*Betweenness centrality* showed the opposite trend showing an overall global decrease in all the learning groups (task 1, 2, and 3) while showing a global increase in the performance group (task 4, *p* < 0.001).

### Local changes in *betweenness centrality*

More specific significant nodal changes for *betweenness centrality* were seen in the explicit SRTT group (task 1) including decreases in the left precental gyrus, the right angular gyrus, left thalamus and right cerebellum crus I, while the implicit SRTT group (task 2) only showed decreases in the left post central gyrus and left caudate (*p* < 0.01). As for the visuomotor learning group (task 3), they also showed a general decrease in the right inferior triangular gyrus and right middle occipital gyrus, cerebellum crus II left (*p* < 0.01), while the performance group (task 4) showed overall increases for this measure in the right precental gyrus and right SMA and a decrease in the cerebullum (*p* < 0.01).

### Global efficiency

Overall *global efficiency* showed a non-significant increase for all the learning groups [task 1 (*p* = 0.23), task 2 (*p* = 0.53) and task 3 (*p* = 0.46)] over all costs while the performance group (task 4) showed a non-significant decrease (*p* = 0.42).

### Modularity

**Figure 12:**
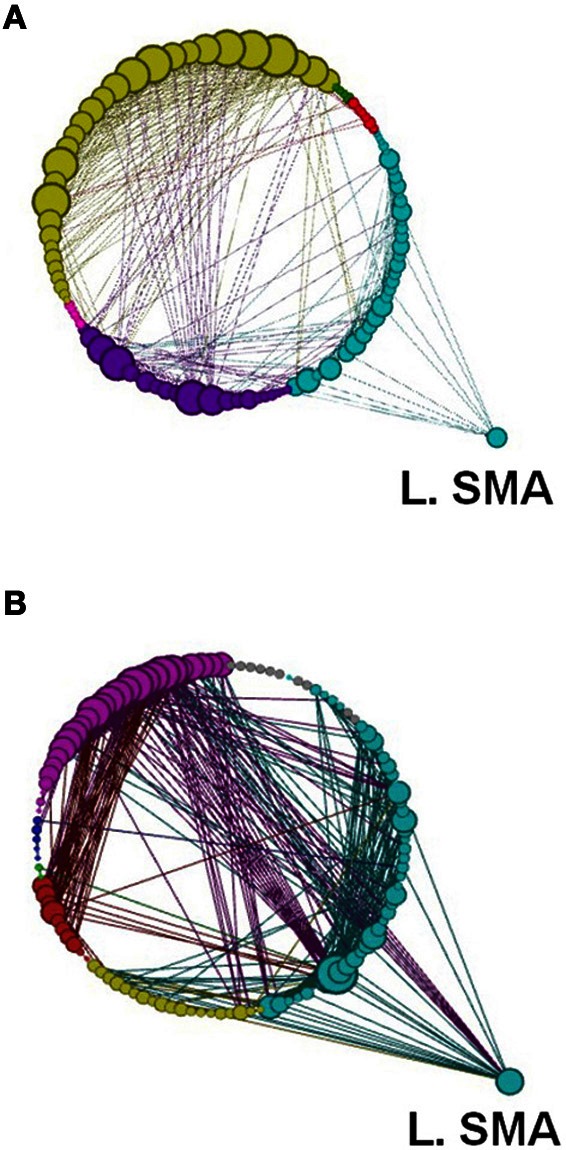
**A depiction of network modularity for both (A) REST 1 and (B) REST 2 produced by the Louvain method for community detection and visualized by the force directed Circular Graph algorithm at a threshold of *r* = 0.6 following the explicit SRTT (task 1).** This figure also highlights the increased connection of Left SMA in Rest 2 and the node size which is proportional to the degree of the node. Color code for modules are Cyan, Sensory-Motor and Prefrontal; Dark Blue, Fronto-Parietal; Red, Cerebellar. Yellow, Visual and Cerebellar; Green, subcortical.

**Figure 13:**
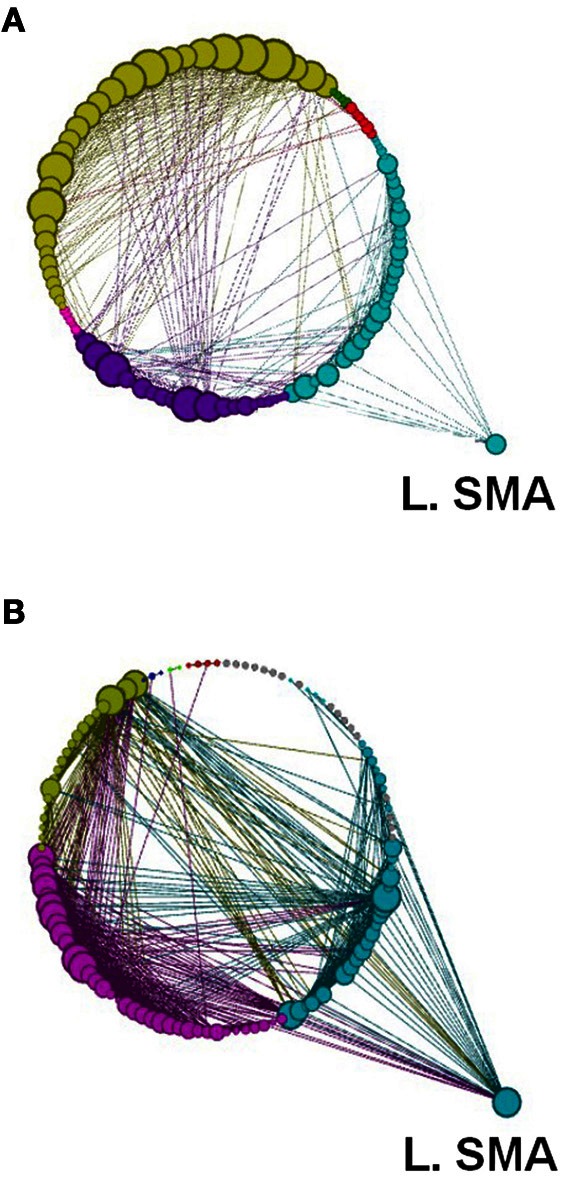
**Network modularity following the implicit SRTT, in the same format as Figure 12**.

**Figure 14:**
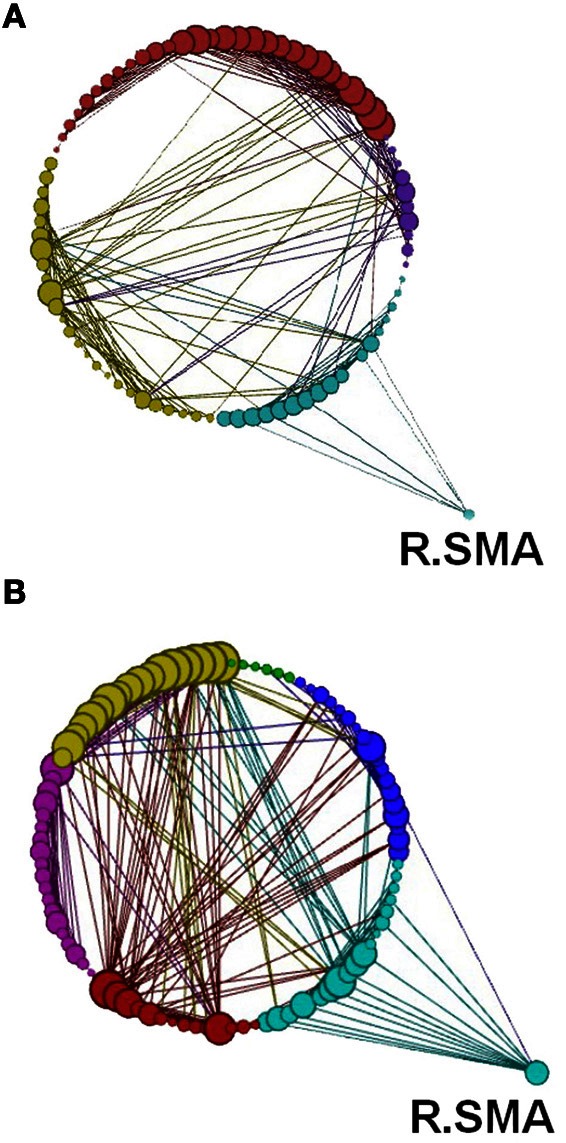
**Network modularity following the visuo-motor adaptation learning task, in the same format as Figure 12.** However, this figure highlights the increased connection to the right SMA in Rest 2. Color code for modules are Cyan, sensory-motor and prefrontal; Dark Blue, fronto-parietal; Red/Pink, cerebellar; Yellow, visual; Green, subcortical.

**Figure 15:**
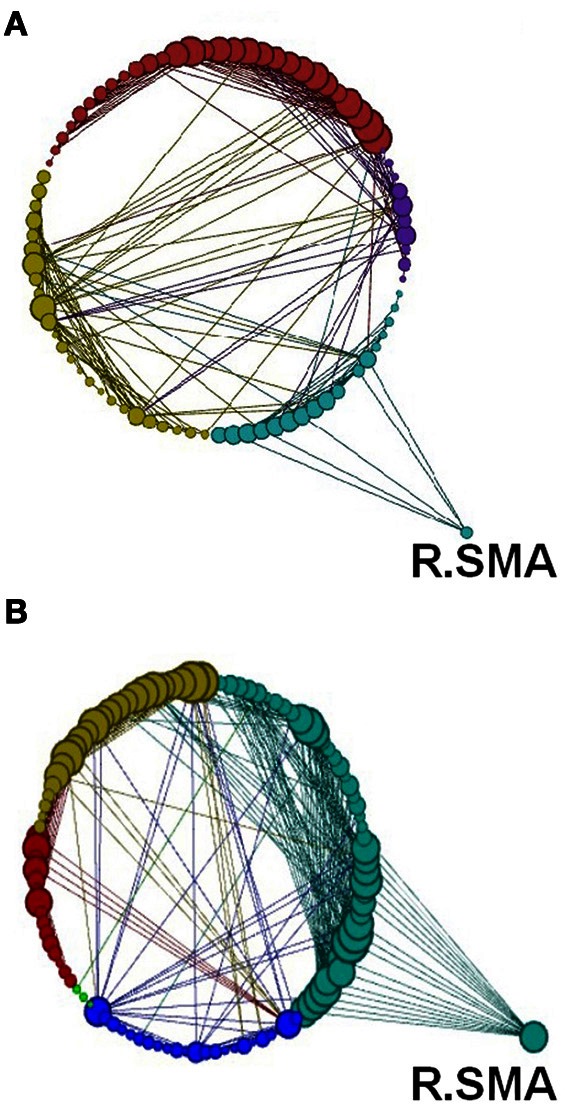
**Network modularity following the visuo-motor performance task, in the same format at Figure 12 and same Color scheme same as Figure 14**.

Another global measure is that of *modularity* in the form of Q value (see Materials and Methods). This showed opposite effects to *global efficiency*, with non-significant decreases for the learning groups and a non-significant increase for the visuo-motor performance group (*p* > 0.05). However, *modularity* exposed a different network distribution between the two SRTT tasks and the two visuo-motor tasks (see Figures 12–15). Additionally, Figure 14 highlights the segregation of cerebellum shown as a separate cluster in the visuo-motor learning task (task 3).

### Participation coefficient

Table 1 shows significant increases in *participation coefficient* for all three motor learning tasks, more specifically the explicit SRTT (task 1) showed widespread cortical increases over frontal, parietal, visual and sub-cortical regions while the implicit SRTT (task 2) increased over sensory motor and sub-cortical regions (*p* < 0.05). The visuo motor learning group (task 3) also showed increases in the frontal cortex, precuneus, temporal gyrus, and multiple areas in the cerebellum, while the performance group showed no significant changes (*p* > 0.05).

**Table 1:** **Brain regions with increased participation coefficients**.

**Anatomical region**	**MNI co-ordinates**	**Task 1**	**Task 2**	**Task 3**
Superior frontal gyrus L.	13, 48, −17	In^*^		In^*^
Superior frontal gyrus R.	−20, 47, −17	In^*^		
Insula L.	34, 8, 0		In^*^	
Hippocampus L.	24, −20, −11	In^*^		In^*^
Parahippocampal gyrus L.	21, −15, −22		In^*^	
Amygdala L.	23, 1, −19			In^*^
Amygdala R.	−27, −1, −20	In^*^		In^*^
Fusiform gyrus L.	29, −40, −21	In^*^		
Supramarginal gyrus R.	−59, −33, 28	In^*^		
Precuneus L.	6, −54, 42			In^*^
Putamen R.	−27, 4, 0	In^*^		
Pallidum R.	−21, 0, −2	In^*^		
Superior Temporal Gyrus R.	−56, 21, 5			In^*^
Crus I L	−35, −67, −29			In^*^
Vermis3	2, −40, −11			In^*^
Vermis6	2, −67, −15			In^*^

Summary of significantly increased brain regions measured in terms of participation coefficients in all three learning tasks at a threshold of r = 0.6. Significant increases are labeled by In^*^.

## Discussion

Our graph analytic results highlight regular patterns in the changes across four resting state functional connectivity data sets. In each case we tested for between pre- and post-motor task changes, showing comparable global topological patterns following the three motor learning tasks, although the different tasks affect different nodes and sub-networks. Moreover, the group performing a “non-learning” visuo-motor task revealed a different global topological pattern in comparison with the three learning groups. The current graph theoretic analysis also emphasizes that motor learning leads to rapid functional reorganization that is maintained during post-learning resting state activity as indicated by emergence of new functional network relationships as a result of training.

Our resting state BOLD results followed an analogous pattern, showing identical changes in all of the key measures aspects of the network topology in comparison to Heitger et al. (2012) graph theoretical results from task-based acquisitions.

Although behavioral differences existed between the tasks, performance differences due to task duration are unlikely to have affected the outcome of the graph analysis results as all the tasks lasted ~10 min.

Task differences showed the expected differential local network changes. Generally a large number of network measures showed that the explicit tasks i.e., task 1 and task 3 affected the prefrontal cortex. These effects were not seen in the implicit condition (task 2). This dissociation between implicit and explicit conditions has also been shown in task based imaging data (Destrebecqz et al., 2005; Fletcher et al., 2005; Ghilardi et al., 2009).

Additionally, our graph analytic RSN results support the hypothesis—based on a recent meta-analysis of task-based fMRI literature—that experience in visuo-motor tasks will show stronger cerebellar changes while the procedural sequence-learning tasks will show more widespread cerebral cortical activity (Hardwick et al., 2012).

This increase in cerebellar activity for the visuo-motor task is particularly distinct in terms of degrees, local efficiency, and participation coefficients highlighting to an increase in both short range local and long distance inter-modular processing. Furthermore, as the SRT tasks were performed with right hand and the visuo-motor were performed with the left a further distinction can be revealed due to handedness these tasks with a greater right hemispheric activation in the case of the visuo-motor tasks.

An added benefit of using graph network measure compared with other standard techniques is that it highlights how different network elements play different roles within the network e.g., some nodes may provide improved local information transfer due to increased local computational demand while other nodes may play a greater role in the longer distance transfer of the information as indicated by *path length* and *betweenness centrality* or may in fact in some cases do both.

As expected the *strength* measure revealed regular enhancement between the pre- and post-exposure measurements for the learning groups, while demonstrating that network connectivity increased most significantly in the explicit SRTT task (see Figure 3). The collective significant increases observed across several graph analytic measures including global *strength*, *degrees*, *correlation coefficients*, and *local efficiency* are all indicative of increased local connectivity in the network. The increases in three of these measures were also observed by Heitger et al. (2012) in task-related BOLD, in participants following a 4-day bimanual coordination training regime with either visual or auditory feedback. Furthermore, the two graph analytic measures of *path length* and *betweenness centrality* confirmed the previously reported decreases following motor learning (Heitger et al., 2012). Reductions in these two measures indicate more direct communication pathways, with fewer intermediate nodes.

These decreased graph measures are likely to affect the global communication patterns, and in support of this, *global efficacy* showed a regular yet non-significant increase across the three learning experiments.

Small-world networks are characterized by a short average path length linking nodes together with a high clustering coefficient (Watts and Strogatz, 1998). This *small worldness* property has been repeatedly shown in both structural and functional neuroimaging over a broad range of spatial and temporal scales detected by a variety of modalities including EEG and MEG (Stam, 2004) and suggests that brain networks are characterized by dense local networks, and by long range connections between these local clusters. However, it has been shown that small-world network properties break down in neuropsychiatric and epileptic patients, making it an important indicator of abnormalities. In our data following motor learning in healthy participants, *small world* properties were maintained but slightly reduced (see Figure 4). This indicates an uneven increase between local and global efficiency as small worldness can also be seen as a ratio between these efficacy measures. It also implies that learning only minimally affects the brains' normal operational boundaries.

Although *small-worldness* provides a useful network topological descriptor for both global and local levels of connectivity, it does not give any information about the sub-network organization, which is instead captured by the *modularity* of the network.

Modularity describes densely connected regions of a community or sub-networks within the same module but sparsely linked to regions in other modules (see Figures 1 and 12–15). Recent studies investigating resting-state BOLD data have found that *modularity* shows meaningful decompositions of the network into related functional sub-networks across a wide range of populations and experimental conditions (Fair et al., 2009; Meunier et al., 2010). Furthermore, *modularity* has been used to highlight associations between functional and structural sub-networks (Hagmann et al., 2008).

Due to these regional increases in density within the same module compared to random graphs of the same size and connection density, there was a positive *Q* value for *modularity* (see Materials and Methods) for rest conditions. However, the decrease in these *Q* values following learning is likely to be due to the increase in the number of nodes participating in a greater number of modules, as indicated by the *participation coefficient*. Intra-modular connectivity therefore showed a significant increase in the number of connector nodes following motor learning (tasks 1, 2, and 3) in the fronto-parietal and hippocampal networks, while the performance group (task 4) showed very minor decreases. *Hub* measures for all three learning tasks were also significantly increased. Among the motor learning tasks the explicit serial reaction time task showed the greatest difference in *connectivity degree, betweeness centrality, mean path length*, and connection *strength*. This was followed by the visuo-motor adaptation task and finally the implicit serial reaction time task. The visuo-motor learning task (task 3) was difficult and very obvious to the participants. Hence it may have considerable explicit components. This suggests that this *hub* outcome could be partly due to the additional areas recruited by these two different explicit tasks (the sequence task 1 and the visuo-motor learning task 3). This is then analogous to the results of Heitger et al. (2012) who also showed that their visual feedback group had a more favorable outcome on all the above measures.

Greater *efficiency* and shorter *path length* of functional links between the nodes of a neural network will probably lead to more rapid transmission times and reduced noise degradation. This increased efficiency also implies that these strengthened functional connections form new “virtual” networks, reducing the need for the equivalent dedicated structural networks, and thus also avoiding the added incremental metabolic costs in terms of modifying physical connections. As such, this may underlie a general brain optimization strategy that may support consolidation of these motor memories, as the brain areas affected following immediate task based changes also play a role in consolidation (Ma et al., 2010; Bullmore and Sporns, 2012; Penhune and Steele, 2012). However, there is likely to be a trade off in longer-term motor learning to be negotiated between generality, efficiency and wiring cost in the optimal configuration of brain networks (Taubert et al., 2011).

## Conclusions

This work has used a number of graph theoretical methods to assess functional connectivity changes in resting state networks following motor learning. Our findings of changes in resting state activity following motor learning tasks are consistent with prior observations of changes in graph metrics that were based on task-related BOLD recordings. This adds further credence to the growing view that resting state network analysis can identify changes in functional connections that are both task-relevant and likely to support longer-term consolidation of these motor memories. An additional finding is that we show for the first time using graph analysis a clear distinction between network changes in groups challenged with motor learning compared to a motor performance group.

Taken together with the other network measures like *local efficiency* these results imply that motor learning results in more direct information transfer across the relevant networks, while motor performance alone either decreased or maintained the status quo.

### Conflict of interest statement

The authors declare that the research was conducted in the absence of any commercial or financial relationships that could be construed as a potential conflict of interest.
